# Supramolecular Complexes of Plant Neurotoxin Veratridine with Cyclodextrins and Their Antidote-like Effect on Neuro-2a Cell Viability

**DOI:** 10.3390/pharmaceutics14030598

**Published:** 2022-03-09

**Authors:** Laura A. Uribe, Sandra Leonardo, Thorbjørn Terndrup Nielsen, Casper Steinmann, Mònica Campàs, Alex Fragoso

**Affiliations:** 1 Departament d’Enginyeria Química, Universitat Rovira i Virgili, Avinguda Països Catalans 26, 43007 Tarragona, Spain; laura.uribe@urv.cat; 2 IRTA, Carretera Poble Nou km 5.5, 43540 Sant Carles de la Ràpita, Spain; sandra.leonardo@irta.cat (S.L.); monica.campas@irta.cat (M.C.); 3 Department of Chemistry and Bioscience, Aalborg University, Fredrik Bajers Vej 7H, 9220 Aalborg, Denmark; ttn@bio.aau.dk (T.T.N.); css@bio.aau.dk (C.S.)

**Keywords:** veratridine, cyclodextrin, neurotoxin, inclusion complex, neuroblastoma 2-a, cell-based assay

## Abstract

Veratridine (VTD) is a plant neurotoxin that acts by blocking the voltage-gated sodium channels (VGSC) of cell membranes. Symptoms of VTD intoxication include intense nausea, hypotension, arrhythmia, and loss of consciousness. The treatment for the intoxication is mainly focused on treating the symptoms, meaning there is no specific antidote against VTD. In this pursuit, we were interested in studying the molecular interactions of VTD with cyclodextrins (CDs). CDs are supramolecular macrocycles with the ability to form host–guest inclusion complexes (ICs) inside their hydrophobic cavity. Since VTD is a lipid-soluble alkaloid, we hypothesized that it could form stable inclusion complexes with different types of CDs, resulting in changes to its physicochemical properties. In this investigation, we studied the interaction of VTD with β-CD, γ-CD and sulfobutyl ether β-CD (SBCD) by isothermal titration calorimetry (ITC) and nuclear magnetic resonance (NMR) spectroscopy. Docking and molecular dynamics studies confirmed the most stable configuration for the inclusion complexes. Finally, with an interest in understanding the effects of the VTD/CD molecular interactions, we performed cell-based assays (CBAs) on Neuro-2a cells. Our findings reveal that the use of different amounts of CDs has an antidote-like concentration-dependent effect on the cells, significantly increasing cell viability and thus opening opportunities for novel research on applications of CDs and VTD.

## 1. Introduction

Veratridine (VTD) is a lipid-soluble alkaloid neurotoxin derived from *Veratrum* plants, which belong to the lily family. It has been used in the past as a drug against arterial hypertension, although its pharmacological use was stopped due to the secondary effects of intoxication. Nowadays, VTD is used as a research tool, given that it acts by targeting the voltage-gated sodium channels (VGSCs) of cell membranes, thus producing a persistent Na^+^ current and increasing intracellular Na^+^ concentration [1]. An important use of VTD in research is enhancing the specificity of neuroblastoma (Neuro-2a) cell-based assays (CBAs) used for the detection, evaluation of toxicity, and study of the mechanisms of VGSC toxins [2]. VGSCs transmit action potentials in neurons, skeletal muscles, and cardiac cells. Unsurprisingly, the alteration in VGSC caused by VTD produces symptoms such as intense retching, bradycardia, hypotension, arrhythmia, loss of consciousness, and seizures. The treatment for VTD poisoning is mainly focused on relieving the symptoms using drugs such as atropine, dopamine, and diazepam [3,4]. Additionally, activated charcoal is used to remove the unabsorbed excess alkaloids [5]. Nevertheless, there is currently no specific antidote that targets the toxin molecule itself.

On the other hand, cyclodextrins (CDs) are cyclic oligosaccharides formed by 6, 7 or 8 glucopyranose units (α, β or γ-cyclodextrin, respectively) forming a truncated cone-like shape that gives them the ability to form host–guest inclusion complexes (ICs). CDs possess a hydrophilic exterior because of the hydroxyl groups that lie in their outer rims. In their inner cavity, the C-H and ether-bond glycosidic oxygens generate an apolar microenvironment where hydrophobic moieties with the right size for the cavity can be encapsulated. Thus, CDs form non-covalent host–guest complexes with a great number of lipophilic molecules [6]. Native CDs can be chemically modified to produce different derivatives such as cationic or anionic CDs, bridged CDs, polymers, etc. These modifications can enhance the stability and selectivity of the formed host–guest ICs [7]. The encapsulation of guests inside the CD cavities changes the physicochemical properties of the resulting guest molecules, for example, an increase in their solubility [8], protection from oxidation [9], protection from visible or UV light degradation [10], hiding flavors or odors [11], and controlling the administration and release of drugs. Their applications include the biotechnology [12], environmental [13], and pharmaceutical industries [14], among many others.

A promising and ever-growing application for CDs is as supramolecular antidotes against toxins. There is research regarding the complexation of CDs with several mycotoxins like alternariol [15], ochratoxin A [16], aflatoxins [17], citrinin [18,19], and zearalenone [20], as well as with some neurotoxins like the rodenticide tetramethylenedisulfotetramine [21] and the insecticide paraoxon [22]. Nevertheless, there is still much room for studying the interactions and applications of CDs with many other neurotoxin molecules that target the VGSC. For example, those that are produced by plants and animals such as VTD, aconitine, grayanotoxins and batrachotoxin, which are small lipid-soluble toxins, or marine toxins produced by algae such as okadaic acid, ciguatoxin, and brevetoxin, which are cyclic polyethers [23].

**Figure 1:**
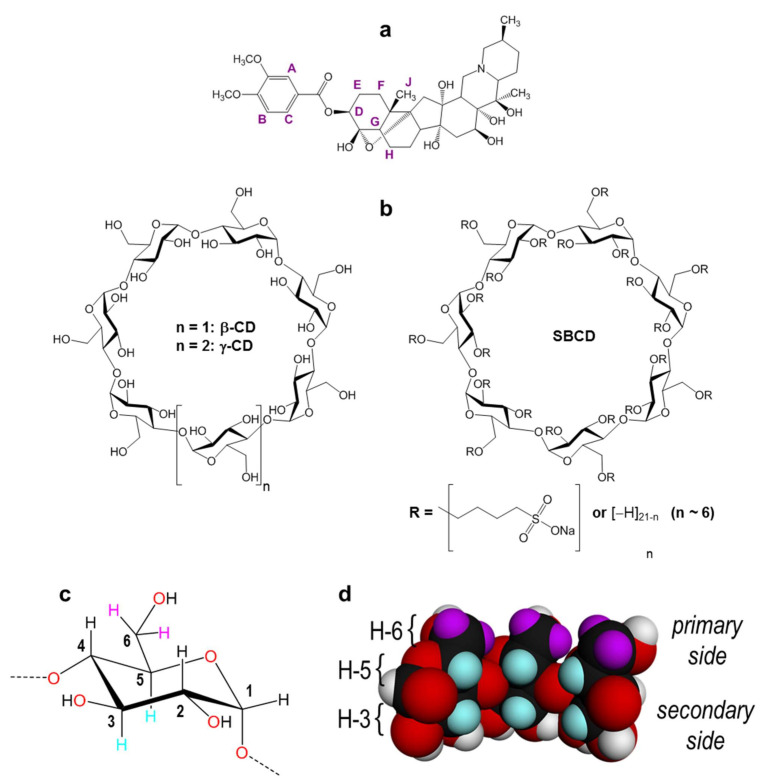
(**a**) Structure of VTD with atom labelling. (**b**) Structure of the cyclodextrin hosts used in this work. (**c**) Atom labelling of the glucopyranose ring. (**d**) Cross-section view from inside the β-CD cavity showing the H-3 and H-5 rims (cyan) and the H-6 atoms located on the border of the primary side (violet).

In this work, we studied the complexation between native β- and γ-cyclodextrin (β-CD and γ-CD) and the anionic derivative sulfobutyl β-cyclodextrin (SBCD) with the neurotoxin VTD for the first time (Figure 1). We performed isothermal titration calorimetry (ITC) studies of the interaction of with β-CD, γ-CD, and SBCD to estimate thermodynamic parameters and the stability constant of the complex in the different CDs. Furthermore, we characterized and confirmed the insertion of VTD inside the CDs cavity using 1D and 2D nuclear magnetic resonance (NMR). A docking and molecular dynamic study confirmed the best CD binders and the most energy stable configuration for the inclusion complex. Finally, with a goal of understanding the effects of the VTD-CD inclusion complexes, we evaluated their toxicity with a cell-based assay (CBA) on Neuro-2a cells.

## 2. Results and Discussion

### 2.1. Isothermal Titration Calorimetry (ITC)

**Figure 2:**
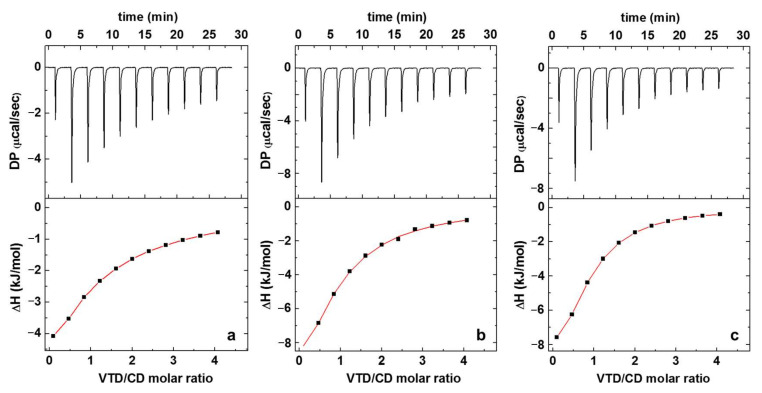
Isothermal titration calorimetry data obtained for the binding interaction of VTD with: (**a**) β−CD, (**b**) γ−CD, and (**c**) SBCD. The upper graphs show the variation of heat released upon injection of 10 μL of each CD. The lower graphs correspond to the binding curve obtained from the integrated heat data. Conditions: [VTD] = 0.2 mM, [CD] = 4 mM, T = 298.2 K.

Calorimetric titrations were carried out in order to verify the formation of host–guest complexes between VTD and CDs. Figure 2 shows the heat evolved and the curve fitting the enthalpy of complex formation for the three studied systems. Table 1 shows the thermodynamic parameters obtained. 

ITC allows one to directly measure the differential heat of binding released or absorbed during the interaction of a binder and a ligand. From the obtained thermogram it is possible to characterize the stoichiometry of the complex (*n*), the equilibrium constant (*K_eq_*), the enthalpy (∆*H*), and entropy (∆*S*) changes, which are related to the Gibbs free energy (∆G) by Equation 1 [24].

(1) $$∆G=RTlnK_{eq}=∆H-T∆S$$

The differential heat measured in ITC depends on a dimensionless parameter (*c*) that determines the shape of the binding isotherm and relates the total host concentration (in this case the CDs) with the equilibrium constant according to Equation 2 [22].

(2) $$c=n\left[CD\right]K_{eq}$$

Values of *c* between 1<c<1000 are considered to be good for accurately estimating the thermodynamic parameters. Ideally, for systems with *K_eq_* values in the order of those commonly observed for CD complexes (10^2^–10^4^ mol^−1^), relatively high concentrations should be used in the experiment to guarantee that the *c* value lies between the appropriate range. However, when working with systems that involve CDs it is important to have in mind that aggregation starts to occur at critical concentrations above 1–2% (m/v) for β-CD, γ-CD, and SBCD [25,26]. This can lead to incorrect measurement of the differential heat of binding since part of the heat is consumed in the aggregation process. This compromise between the CD concentrations and *c* values could be easily attained in the case of the γ-CD and SBCD due to their high solubility. 

As can be seen from Table 1, the formation of inclusion complexes with γ-CD and SBCD is mainly enthalpy driven, since $\left|∆H\right|>\left|T∆S\right|$ [27]. The thermodynamic parameters obtained for CD complexes are a result of the contributions of the release of water molecules contained inside the CD cavities driven by the occurrence of van der Waals and hydrophobic interactions between the host and the guest [28]. The large exothermic effect seen in the negative enthalpy values of γ-CD and SBCD indicate that their interaction with VTD is mostly driven by van der Waal forces, rather than by hydrophobic interactions [27], as observed in other CD complexes [24,29,30].

On the other hand, γ-CD and SBCD have *K_eq_* values of 7200 M^−1^ and 8200 M^−1^, respectively, in comparison with 1500 M^−1^ for β-CD (Table 1), indicating that the two first CDs form more stable inclusion complexes with the VTD toxin. This can be attributed to the larger cavity size in the case of γ-CD in comparison to β-CD, where the VTD guest can potentially fit better. In the case of the anionic SBCD derivative, the diameter of the cavity is the same as β-CD, but the cavity is enlarged due to the sulfobutyl arms chemically attached through ether bonds. This longer cavity also helps to accommodate the VTD toxin. Since the *pK_a_* of VTD is 9.5 [31], at pH 6 the molecule is protonated and electrostatic interactions between the SO_3_^−^ groups and the NH^+^ group of VTD also help to stabilize the inclusion complex, resulting in a higher *K_eq_* for VTD-SBCD than in the case of the VTD-γ-CD. Finally, β-CD has the lowest affinity for the VTD toxin. This is expected, since a weaker inclusion complex is formed due to the smaller cavity size where the VTD toxin can fit only partially, resulting in the smaller *K_eq_* value.

### 2.2. NMR Experiments

**Figure 3:**
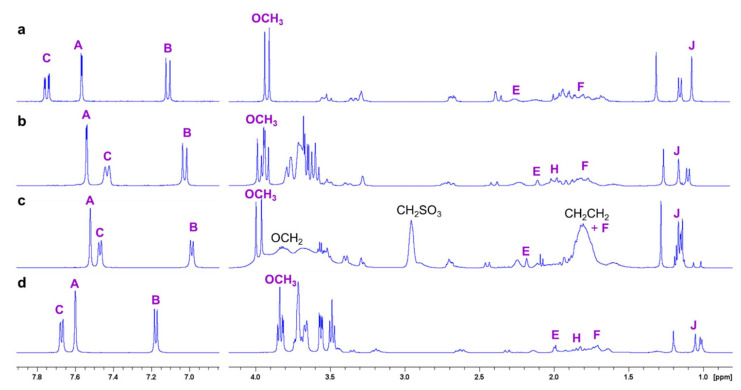
High frequency (left) and low frequency (right) regions of the ^1^H-NMR spectra (0.1% DCl in D_2_O, 500 MHz, 298 K) of: (**a**) VTD, (**b**) VTD:β-CD, (**c**) VTD:SBCD, and (**d**) VTD:γ-CD. The VTD protons are identified in violet and the SBCD spacer protons in black (see Figure 1). For the assignment of the other cyclodextrin protons please see Figure 4, Figure 5 and Figure 6.

VTD is the 3,4-dimethoxybenzoate ester of a 6-ring steroidal structure. The molecule thus features two well-distinguished parts that should interact in different ways with the CD hosts. The strong overlapping of some steroid protons with those of the CD hosts in the ^1^H-NMR spectra prevented an accurate analysis of the effect of VTD on H-3 and H-5, which point towards the cavity and are usually the most affected by guest inclusion (Figure 3). However, analysis of the aromatic part of the spectra gave some hints on the possible inclusion geometry. Figure 3 (left) shows the ^1^H-NMR spectra of the aromatic protons of VTD and their inclusion complexes with the three studied CDs. In all cases, the peaks of the VTD-CD complexes are shifted with respect to free VTD, indicating inclusion of the aromatic moiety in the CD cavities. In the case of β-CD and SBCD, all proton signals are shielded, with proton C showing the highest displacement (~0.3 ppm). The shift to lower frequencies of protons A and B was higher for SBCD (0.04 and 0.13 ppm, respectively) than for β-CD (0.02 and 0.06 ppm, respectively), suggesting a stronger interaction of the aromatic moiety with the anionic host, consistent with the ITC results. In the case of γ-CD, the aromatic resonances were less affected by the inclusion. Interestingly, protons A and B are slightly shifted to higher frequencies with respect to VTD, suggesting that these protons are close to oxygen atoms outside the cavity of the host [32,33]. This is an indication that the steroid part is deeply included in the cavity of γ-CD due to its larger size caused by the 3,4-dimethoxybenzoate group protruding from the primary side. 

**Figure 4:**
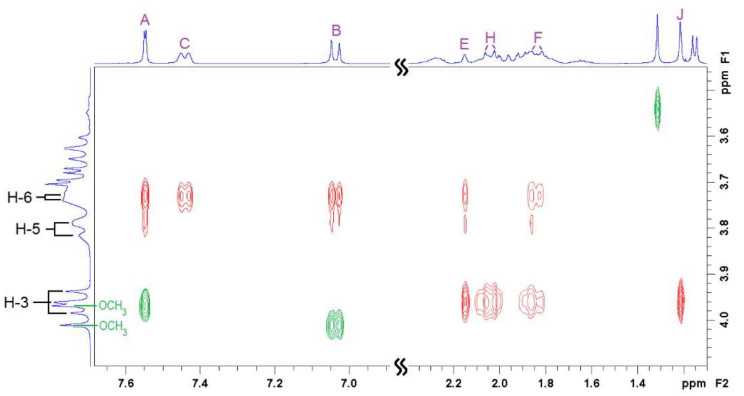
Two-dimensional ROESY NMR spectrum of the 1:1 VTD:β-CD inclusion complex showing intermolecular (**red**) and intramolecular (**green**) cross-peaks. See Figure 1a,c for atom labelling.

**Figure 5:**
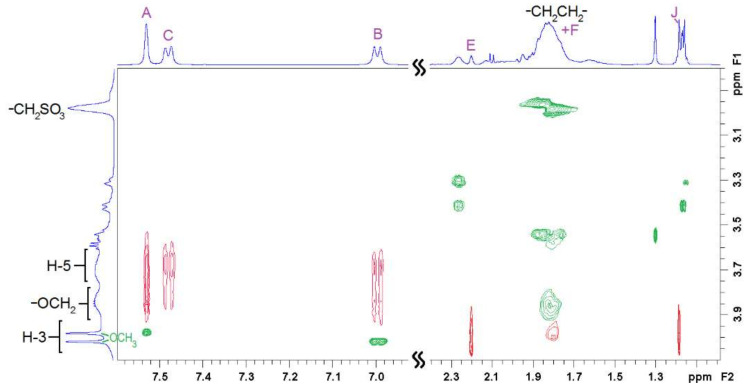
Two-dimensional ROESY NMR spectrum of the 1:1 VTD:SBCD inclusion complex showing intermolecular (**red**) and intramolecular (**green**) cross-peaks. See Figure 1a,c for atom labelling.

**Figure 6:**
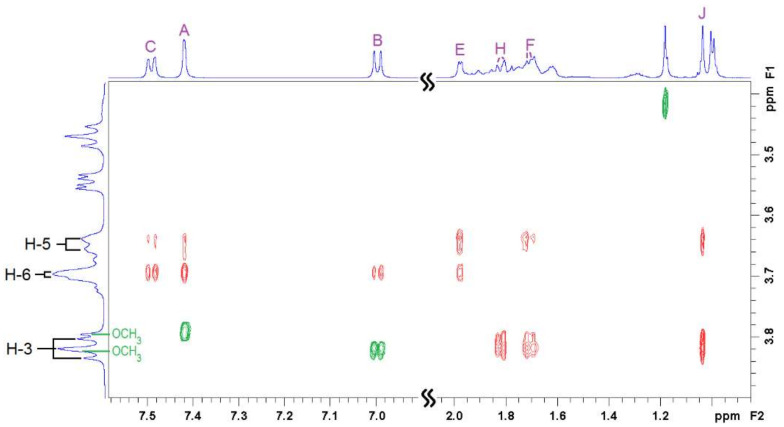
Two-dimensional ROESY NMR spectrum of the 1:1 VTD:γ-CD inclusion complex showing intermolecular (**red**) and intramolecular (**green**) cross-peaks. See Figure 1a,c for atom labelling.

Two-dimensional ^1^H-^1^H rotational Overhauser enhancement experiments (ROESY) are very useful tools to study the solution geometry of inclusion complexes with CDs. They provide information on the through-space proximity (typically 3–4 Å) of host protons and the guest parts involved in the supramolecular complexation in the form of cross-peaks, with intensity proportional to the proximity of the protons involved. Figure 4, Figure 5 and Figure 6 show the ROESY spectra of the studied 1:1 VTD inclusion complexes in D_2_O. 

The ROESY spectrum of the VTD:β-CD inclusion complex (Figure 4) shows cross-peaks between the aromatic protons and H-5. This indicated that VTD enters the cavity though the wider secondary side, with the aromatic ring residing close to the primary side. This was further confirmed by the presence of strong cross-peaks between protons E, F, and H, and the methyl group J of VTD and H-3. Protons E and F also showed weaker cross-peaks with H-5. These protons correspond to the steroid ring closer to the 3,4-dimethoxybenzoate group, indicating that this part of the VTD molecule also enters the cavity. 

In the case of the VTD:SBCD inclusion complex (Figure 5), the ROESY spectrum showed a similar cross-peak pattern to VTD:β-CD and thus indicates a comparable inclusion geometry. Besides the steroid/H-3 and the aromatic/H-5 cross-peaks, the spectrum reveals spatial vicinity between the aromatic protons and the OCH_2_ protons of the sulfobutyl group located on the primary side [34], although we could not detect cross-peaks with the VTD methoxy groups due to signal overlap. No interactions with the other sulfobutyl methylene groups were observed. 

The ROESY spectrum of VTD:γ-CD also reveals an inclusion geometry in which the aromatic ring sits farther to the primary side of the γ-CD cavity as compared with β-CD. In this case, the aromatic protons show weaker interactions with H-5 but stronger with the H-6 protons that lie on the border of the cavity. This is in agreement with the signal displacements that were observed on the 1D ^1^H-NMR spectrum. Protons E, H and F also showed cross-peaks with H-3 and H-5, confirming the inclusion of the steroid part and a deeper penetration in the cavity. Interestingly, methyl group J also showed a strong signal with both H-3 and H-5, in contrast to β-CD in which this group lied closer to H-3. The differences in the relative position of the VTD guest in the β-CD and γ-CD cavity is mainly due to the larger size of γ-CD, that accommodates the bulky steroidal part better.

### 2.3. Molecular Simulations

**Figure 7:**
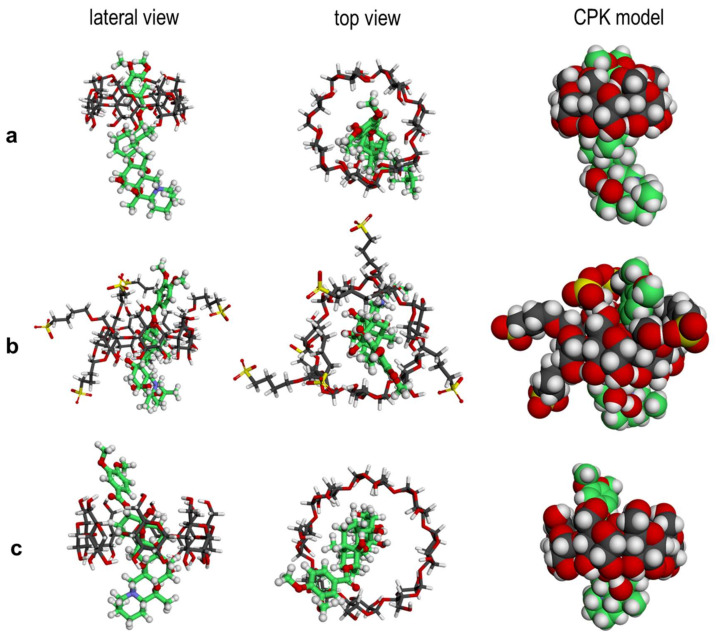
Three-dimensional energy-minimized structures obtained by molecular docking of the inclusion complex between VTD and (**a**) β-CD, (**b**) SBCD, and (**c**) γ-CD. The C atoms of VTD have been highlighted in green.

Molecular docking is a widely used tool that aids to understand the geometries and interactions involved in the formation of inclusion complexes and often complements the experimental results [35]. From the simulation results presented in Table 2 we observe that all three CDs are predicted to be potential binders for the VTD. From docking, we obtained −25.9 kJ/mol, −26.7 kJ/mol and −5.6 kJ/mol for SBCD, γ-CD and β-CD, respectively. These results are in agreement with the experimental results obtained by ITC, where SBCD and γ-CD are stronger binders for VTD than β-CD. From the 3D structures we can see that the best pose for the VTD:β-CD complex (Figure 7a) is one where the VTD molecule is not completely introduced inside the β-CD cavity. The aromatic ring is located inside the cavity and most of the steroid part of the molecule lies outside the cavity. In the case of SBCD (Figure 7b), although it is a β-CD derivative, the host has an elongated cavity because of the presence of the sulfobutyl ether arms that create more space for the guest, resulting in more penetration of VTD. Additionally, its anionic character may help to further stabilize the inclusion complex by electrostatic interactions with the protonated nitrogen atom present in the steroid part. On the other hand, the best pose of the VTD complex with the larger γ-CD host (Figure 7c) shows that the guest is deeply introduced inside the cavity and that the aromatic ring actually protrudes from the primary side. This results in a sort of pseudorotaxane structure in which the host cavity mainly interacts with the steroid part. As can be seen, the docking results are in good agreement with the 2D-ROESY NMR experiments, confirming the validity of this tool to understand inclusion complexation in cyclodextrins. 

Docking studies involve several simplifications: the simulations are not dynamic and normally they do not use an explicit solvent [36]. They are a good tool to quickly estimate the best binding pose for an inclusion complex [37] but they should be complemented with more thorough methods such as MD which, in fact, uses explicit water molecules and considers a dynamic component of the inclusion complexes. Hence, the docking simulations were complemented with a molecular dynamics approach (trajectory videos available in the Supplementary Materials). The free energies of VTD binding with β-CD and γ-CD obtained with MM/GBSA [38] were −1.6 ± 1.3 kJ/mol and −18 ± 3 kJ/mol, respectively. An interesting result was seen in the MD of the γ-CD complex, where at the start of the trajectory simulation VTD was in fact not docked inside of the γ-CD cavity. As the simulation time elapsed, it could be seen how the molecule became incorporated inside the cavity, confirming again that this is the most energy stable configuration for the molecules in solution. The computed MM/GBSA ∆G° values also match both with the docking scores and the ITC data, confirming that γ-CD is a much better binder than β-CD. The underestimation of ∆G° for the β-CD complex (−1.6 ± 1.3 kJ/mol) is most likely due to the geometry of the inclusion complex, in which most of the steroid part of VTD is outside the cavity and not complexed inside the cavity and thus exposed to the solvent (see Figure 7a). 

### 2.4. Cytotoxicity Evaluation

**Figure 8:**
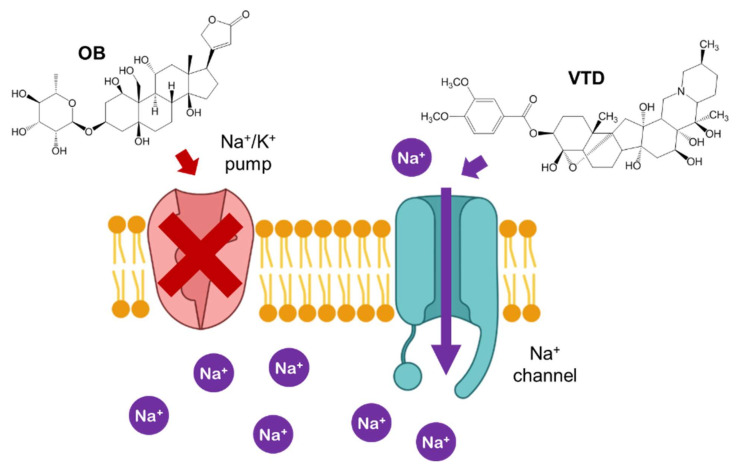
Structure of OB and effect of VTD and OB in the Na^+^ cell transport.

The VTD toxicity on Neuro-2a cells was evaluated with a CBA [39]. In this CBA, ouabain (OB), a toxic cardiac glycoside that inhibits the Na^+^/K^+^ pump [40], is added for the specific and sensitive detection of VTD, since it prevents cells from counteracting the increasing sodium intracellular concentrations produced by VTD exposure [41,42]. The exposure to OB sensitizes the cells and usually results in a slight decrease of the cell’s viability (Figure 8).

**Figure 9:**
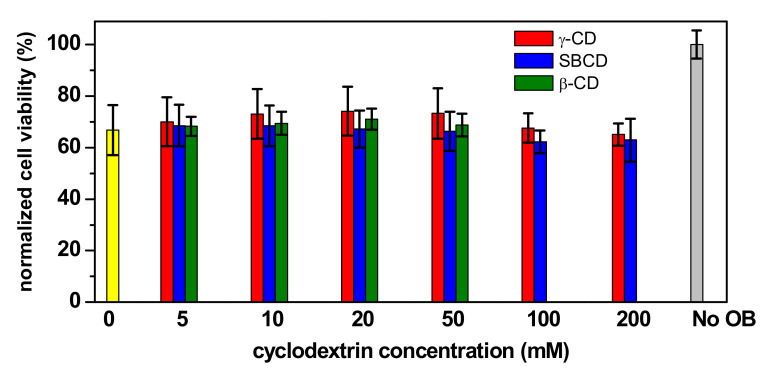
Normalized cell viability of Neuro-2a cells pre-treated with 0.4 mM ouabain (OB) in the absence (**yellow**) and in the presence of γ-CD (**red**), SBCD (**blue**) and β-CD (**green**) at different concentrations. The control in absence of OB is the grey bar.

In our experiments, the pre-treatment of the Neuro-2a cells with 0.4 mM OB resulted in around a 30–40% decrease in cell viability (Figure 9). The effect of CDs on the Neuro-2a cells was simultaneously evaluated. Cells were exposed to different CDs concentrations up to 50 mM, in the case of β-CD (higher concentrations could not be tested because of the low solubility of this CD), and up to 200 mM for γ-CD and SBCD. Cell viabilities around 60–70% were obtained in the absence (0 mM CD) and in the presence of all CDs and at all studied concentrations, demonstrating that CDs are harmless to Neuro-2a cells. On the other hand, this experiment also suggests that the CDs do not form host–guest complexes with OB. This is not surprising, since OB is the glycoside of a pentahydroxylated steroid and thus a highly polar and hydrophilic molecule with little or no affinity for the CDs.

**Figure 10:**
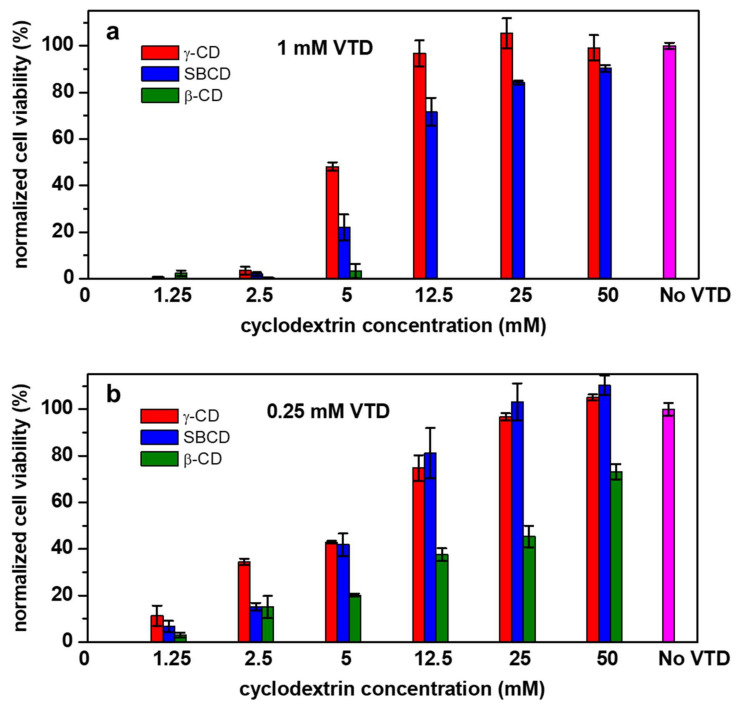
Normalized cell viability of Neuro-2a cells pre-treated with 0.4 mM ouabain (OB) after the incubation with (**a**) 1 mM and (**b**) 0.25 mM VTD in the absence (0 mM) and in the presence of γ-CD (red), SBCD (blue) and β-CD (green) at different concentrations. The control in absence of VTD is the magenta bar.

Neuro-2a cells were then exposed to different VTD and CDs concentrations, in the presence of OB, to test if the formation of inclusion complexes between VTD and the studied CDs resulted in an inhibition of VTD toxicity. In the absence of CDs, the addition of 1 and 0.25 mM VTD caused around 100% cell mortality (0% cell viability) (Figure 10). For 1 mM VTD, cell viabilities increased with increasing CD concentrations for γ-CD and SBCD, reaching around 100% of the cell viability and protecting the Neuro-2a cells from the VTD toxicity. (Figure 10a). However, β-CD was unable to protect the Neuro-2a cells at this VTD concentration. These results agree with the higher stability constants that were determined for the γ-CD and SBCD complexes in comparison to those for β-CD. The results also show that the cell culture medium is not affecting the ability of CDs to complex VTD and highlight the selectivity of the CDs for VTD and not towards other molecules present in the medium.

Experiments were then performed using 0.25 mM VTD. In this case, all CDs were able to neutralize the toxic effect of VTD totally or partially, again in a concentration-dependent manner (Figure 10b). As expected, lower CDs concentrations are needed to inhibit the VTD toxicity. In the case of β-CD, even though a higher concentration (compared to γ-CD and SBCD) is required to inhibit the VTD toxicity, 70% cell viability recovery is achieved at the highest tested β-CD concentration of 50 mM. Therefore, the three studied CDs have antidote-like characteristics, which depend on the type of CD as well as on VTD and CD concentrations. 

## 3. Materials and Methods

All reagents used were of analytical grade and used without further purification. β-CD and γ-CD were obtained from Wacker Chemie AG (Germany). Sulfobutyl β-cyclodextrin was a gift from Cyclolab Ltd. (Hungary). Veratridine (>90% HPLC), D_2_O (99.99% D) and DCl (35%, 99.9% D) were purchased from Sigma Aldrich. The pH 6 buffer was prepared using tris base from Sigma Aldrich and the pH was adjusted using 1 M HCl. At this pH, concentrated stock solutions of VTD could be prepared without solubility limitations. The Neuro-2a cell line was obtained from the American Tissue Culture Collection (ATCC), batch CCL131. 

### 3.1. Isothermal Titration Calorimetry

ITC was performed using a Microcal VP-ITC isothermal titration calorimeter (Microcal Inc., Northampton, MA, USA) at 298.2 K and atmospheric pressure. The instrument was calibrated electronically. The data were acquired with a computer software provided by Calorimetry Sciences Corp and analyzed using the one-site model. VTD/CD binding experiments were performed by injecting 10 µL aliquots with 240 s of separation of a CD solution (4 mM) into the sample cell containing VTD solution (200 μM). All experiments were performed with constant stirring (200 rpm) driven by a stepping motor coupled to the isothermal titration calorimeter. A 20 mM pH 6 tris buffer was used to prepare the solutions and all the solutions were degassed before the titration experiment. The CD concentrations for the experiment were chosen in order to work below the critical aggregation concentration (cac) of each CD [26,43], to ensure that the measured enthalpy change represents the complex formation without contributions from a simultaneous dissolution of the CD aggregates. In control experiments, 10 µL aliquots of a CD solution (4 mM) were injected into the sample cell containing tris buffer without the VTD toxin. 

### 3.2. NMR Spectroscopy

The ^1^H-NMR and 2D ^1^H-^1^H ROESY spectra were recorded in D_2_O containing DCl 0.1% (*v*/*v*) at 400 MHz in a Varian NMR System 400 at 298 K using a 1:1 VTD:CD molar ratio for each cyclodextrin (β-CD, γ-CD or SBCD). All signals were referenced to internal HDO (4.79 ppm). The ROESY spectra were acquired with a mixing time of 400 ms and a relaxation delay of 1.8 s. Proton resonances of VTD, the pure CDs, and the inclusion complexes were assigned with the aid of standard COSY and HSQC experiments on the same solutions. 

### 3.3. Molecular Simulation

#### 3.3.1. Structure Preparation

Preparation of the structure and analysis were both carried out with Maestro v 9.1, (Schrödinger LLC, New York, NY, USA, 2010). The 3D structures of β-CD and γ-CD were retrieved from the Protein Data Bank. SBCD used has an average degree of substitution of approximately 6 sulfobutyl ether chains, as determined by ^1^H-NMR, which are randomly substituted in the β-CD hydroxyl groups. The actual molecular configuration of the molecule is unknown and other groups have reported the building of isomers for in-silico docking tests [44,45]. The SBCD isomer used here was built with the Maestro Builder module. The glucopyranose units 1 and 5 (counted from *n* = 1 in Figure 1b) have a double substitution of the SBE side chains at C-2 + C-6 and C-3 + C-6 respectively. As for glucopyranose units 2 and 7, both have mono-substitutions of the SBE side chains at C-6. We performed a Conformational Search in Maestro to find the energy minimized structure for the built SBCD isomer using an OPLS 3 force field [46] and water as solvent. For the VTD ligand, the structure was retrieved from the Cambridge Crystallographic Data Centre (ID: BUWMIP, deposition number 1117590) and used for the docking studies [47].

#### 3.3.2. Docking Studies

We performed docking of VTD into the three CDs with Glide [48,49] to obtain free energies of binding. A grid for each host was generated with the center of mass of each CD used to define the box where the VTD was to be docked. For all docking simulations, rigid docking was applied and Glide XP was used in Extra Precision mode [50], which uses explicit water molecules for the docking. This better simulates the displacement of high energy water from the lipophilic cavities, thus obtaining more reliable docking scores [36].

#### 3.3.3. Molecular Dynamics Experiments

We obtained free energies of binding for the inclusion complexes of VTD and the CDs using the molecular mechanics (MM) generalized Born (GB), and surface area (SA) continuum solvation model (MM/GBSA) [38] available in the AMBER program package [51]. The starting structures were obtained by manually placing the VTD ligand inside the cavity of the β-CD and γ-CD. Free energies of binding with SBCDs were not performed, due to a lack of suitable parameters. The VTD ligand was parametrized with the general amber force field (GAFF) [52]. The CDs were parametrized with the GLYCAM-06 force field [53]. The entire inclusion complex was solvated in a cubic box of TIP3P water [54]. The side of the box was at least 14 Å away from the inclusion complex. We first minimized the system with the conjugate gradient method for 1000 steps, followed by slowly heating the system with NVT from a temperature of T = 0 K to T = 300 K using Langevin dynamics [55] with a collision frequency of 2 ps^−1^. This was followed by equilibrating the pressure in the NPT ensemble using a Berendsen barostat [56] keeping the pressure at 1 atm. After equilibration, a 100 ns production run was performed to obtain the trajectory and on completion of the simulation, the free energies of binding were computed using the MM/GBSA model.

### 3.4. Neuro-2a Cell Viability Experiments

Neuro-2a cells (ATCC, CCL131) were maintained in 10% fetal bovine serum (FBS) RPMI medium (Sigma-Aldrich, St. Louis, MO, USA) at 310 K in a 5% CO_2_ humidified atmosphere (Binder, Tuttlingen, Germany) [42]. For the experiments, cells were cultured in a 96-well microplate in 5% FBS RPMI medium at an approximate density of 34,000 cells per well for 24 h. A stock solution of VTD (1 mM) was prepared in MilliQ^®^ water and adjusted to pH 2 for solubilization. Stock solutions of CDs (50 mM β-CD, 200 mM γ-CD, and 200 mM SBCD) were prepared in PBS. Prior to exposure to VTD and CDs, one half of the microplate was treated with 0.4 mM ouabain (OB, Sigma-Aldrich). Then, 10 μL of VTD and 10 μL of CDs at different concentrations were added into the wells both with and without the OB treatment (as a control to evaluate CDs toxicity) and incubated for 24 h. Each well had a final pH value of 6. Cell viability was assessed in triplicate experiments using the colorimetric [3-(4,5-dimethylthiazol-2-yl)-2,5-diphenyltetrazolium] MTT assay [57]. Absorbance values were read at 570 nm using an automated multi-well scanning spectrophotometer (Biotek, Synergy HT, Winooski, VT, USA). The cell viability values were normalized with respect to the viability of the control without OB treatment.

## 4. Conclusions

We have successfully proven the formation of inclusion complexes between the alkaloid neurotoxin VTD and native β-CD and γ-CD as well as the anionic β-CD derivative SBCD. The equilibrium constants were estimated to be 1500 M^−1^, 7200 M^−1^ and 8200 M^−1^ for β-CD, γ-CD and SBCD, respectively, making the γ-CD and the anionic SBCD the most stable hosts. The ^1^H-NMR and ^1^H-^1^H ROESY experiments confirmed the incorporation of VTD in each of the CDs’ cavities and the most stable orientation of the molecule inside the CDs was elucidated by performing docking and molecular dynamics simulations. In vitro studies showed that the three studied CDs have antidote-like effects against the VTD toxicity, protecting Neuro-2a cell viability to different extent, depending on the CD type as well as the CD and VTD concentrations. To the best of our knowledge, this is the first study of the interactions between the VTD neurotoxin and CDs and opens the door to further studying of the role of CDs with other lipid toxins. 

## Figures and Tables

**Table 1:** Stability constants (*K_eq_*), standard enthalpy changes (∆*H°*), Gibbs’s binding energy (∆*G°*), and entropy changes (∆*S°*) for the formation of VTD complexes with three CD hosts (γ-CD, SBCD, and β-CD) in tris buffer solution pH 6 at 298.2 K.

	*n*	*K_eq_* (M^−1^)	∆*H°* (kJ/mol)	∆*G°* (kJ/mol)	∆*S°* (kJ/mol)
**β-CD**	0.7 ± 0.2	1500 ± 70	n.d.	−18.1 ± 0.1	n.d.
**γ-CD**	0.95 ± 0.03	7200 ± 100	−16.7 ± 0.2	−22.1 ± 0.1	0.21 ± 0.01
**SBCD**	0.94 ± 0.02	8200 ± 60	−14.5 ± 0.1	−22.4 ± 0.1	0.31 ± 0.01

n.d.: could not be determined accurately.

**Table 2:** Gibbs binding energy (∆G°) values obtained by ITC and molecular dynamics (MD) and docking scores for the formation of inclusion complexes between VTD and the studied CD hosts.

	*K_eq_* (M^−1^)	∆*G°* (kJ/mol) ITC	∆*G°* (kJ/mol) MD	*Docking Score* (kJ/mol)
**β** **-CD**	1500 ± 70	−18.1 ± 0.1	−1.6 ± 1.3	−5.6
**SBCD**	8200 ± 60	−22.4 ± 0.1	n.d.	−25.9
**γ** **-CD**	7200 ± 100	−22.1 ± 0.1	−18 ± 3	−26.7

n.d.: not determined. SBCD molecule not available in the literature for MD studies.

## Data Availability

All data have been included in the article.

## Supplementary Materials

The following supporting information can be downloaded at: https://www.mdpi.com/article/10.3390/pharmaceutics14030598/s1. Video S1: Molecular dynamics simulation of VTD/β-CD complex. Video S2: Molecular dynamics simulation of VTD/γ-CD complex.
